# Draft genome sequences of *Salmonella* recovered from organic and conventional retail chickens in Maryland, USA

**DOI:** 10.1128/mra.01035-24

**Published:** 2025-02-21

**Authors:** Anuradha J. Punchihewage-Don, Zhao Chen, Jianghong Meng, Salina Parveen

**Affiliations:** 1 Food Microbiology and Safety Laboratory, Department of Agriculture, Food and Resource Sciences, University of Maryland Eastern Shore, Princess Anne, Maryland, USA; 2 Joint Institute for Food Safety and Applied Nutrition, Center for Food Safety and Security Systems, University of Maryland, College Park, Maryland, USA; University of Maryland School of Medicine, Baltimore, Maryland, USA

**Keywords:** *Salmonella*, antibiotic resistance, virulence genes, plasmids, organic, chicken, conventional, draft genome sequences, *Salmonella* pathogenicity islands, multidrug resistance

## Abstract

We report the draft genome sequences of 94 multidrug-resistant *Salmonella* isolates (*S*. Infantis, Enteritidis, and Typhimurium) from organic and conventional retail chickens in Maryland, USA (average genome size = 4.97 Mb; guanine–cytosine content = 52.12%). These isolates harbored diverse antimicrobial resistance and virulence genes, *Salmonella* pathogenicity islands, and plasmids, highlighting potential public health risks.

## ANNOUNCEMENT

*Salmonella* Infantis, Enteritidis, and Typhimurium are key serovars of *Salmonella enterica* globally associated with human foodborne outbreaks linked to chickens (1, 2). The majority of those recovered *Salmonella* isolates from outbreaks were shown to be antimicrobial resistant (AMR) to medically important antimicrobials, which are crucial for human and veterinary medicine (2, 3). In the United States alone, *Salmonella* causes more than a million cases of illness, 26,500 hospitalizations, and over 400 deaths annually (4). Poultry is recognized as a major source of *Salmonella* among various food sources (5–7).

These sequenced *Salmonella* isolates were recovered from organic and conventional whole chickens purchased from a retail store in Maryland, USA using United States Department of Agriculture (USDA)-Food Safety and Inspection Service’s recommended whole broiler carcasses enrichment method, as detailed in our previous studies (8–10). Over a 1-year period of monthly sampling (*n* = 480; organic = 240 and conventional = 240), 213 *Salmonella* isolates were recovered and subjected to antimicrobial susceptibility testing (AST) following the guidelines of the Clinical and Laboratory Standards Institute (8, 11). The serovars were serotyped at the USDA National Veterinary Services Laboratories (8, 12). Based on the AST results, only the multidrug-resistant strains [*n* = 94; Infantis (*n* = 71), Enteritidis (*n* = 13), and Typhimurium (*n* = 10)] were subjected to whole-genome sequencing (WGS).

The confirmed *Salmonella* isolates were re-streaked on the tryptic soy agar plates and incubated for 24 h at 37°C. The DNA extraction from *Salmonella* isolates was performed using the DNeasy Blood and Tissue Kit (QIAGEN Inc., Valencia, CA, USA), following the manufacturer’s protocol with slight modifications. During pre-treatment, a few *Salmonella* colonies were combined with 40 µL of proteinase K and incubated in a 56°C water bath for 90 min, with vortexing every 20 min. An Illumina MiSeq platform (Illumina Inc., San Diego, CA, USA) was used for WGS. DNA (1 ng) was utilized for library preparation with the Nextera XT DNA Library Preparation Kit (Illumina Inc., San Diego, CA, USA) according to the manufacturer’s protocol. Libraries were then sequenced using the MiSeq Reagent Kit v3 (600 cycles) with 2 × 300 bp paired-end reads on the MiSeq system.

After WGS, the resulting FASTQ files were trimmed using Trimomatic 0.38.1 and assembled employing SPAdes 3.12 on the GalaxyTrakr platform (v 21.09) (13). Quast 5.2.0 was used for quality assessment of the MiSeq data. The genomes were annotated with default parameters used, unless otherwise specified. SeqSero2 1.1.1 was used to confirm serotypes (14). Staramr 0.9.1 was employed to detect AMR genes (15). ABRicate 1.0.1 integrated with the Virulence Factors Database was used to screen for virulence genes (VG) (16). SPIFinder 2.0 was used to identify *Salmonella* pathogenicity islands (SPIs) (17).

**Fig 1:**
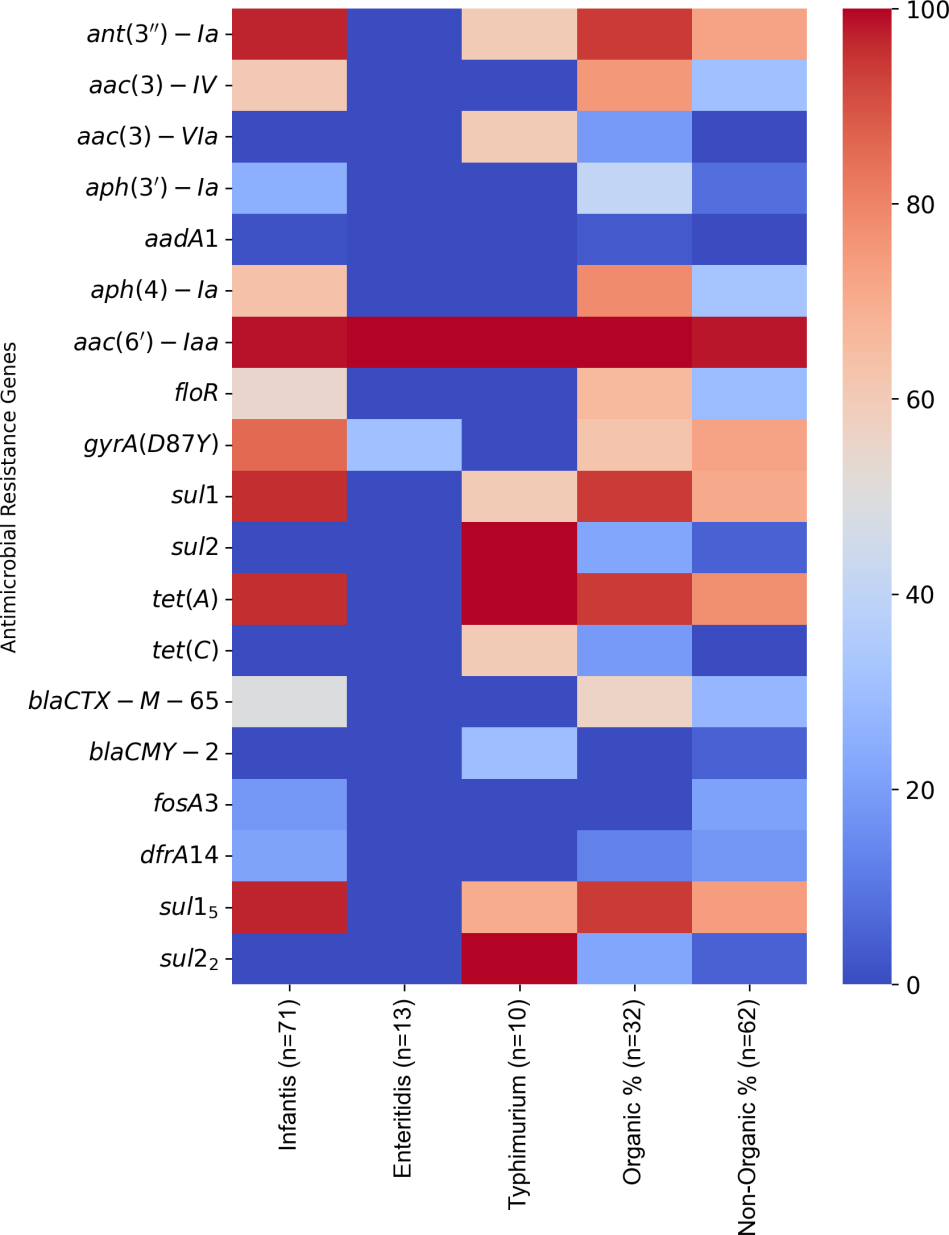
Prevalence of antimicrobial resistant genes among *Salmonella* serovars recovered from both organic and non-organic chickens. The heatmap was generated using Python (v3.12), with “seaborn” for heatmap visualization and “matplotlib” for figure customization.

The genome assembly yielded an average total length of 4.97 Mb with a guanine–cytosine (GC) content of 52.12% (Table 1). The genome comprised an average of 409 contigs with an *N*_50_ value of 110,317 bp. The assembly quality had an L50 value of 88.93. The minimum number of contigs was 37, and no *N’*s were detected per 100 kbp. These isolates carried various AMR (Fig. 1), VG, SPIs, and plasmids, emphasizing potential public health risks.

**TABLE 1:** BioSample, assembly, and GenBank accession numbers for *Salmonella* isolates deposited in BioProject PRJNA1080051

Strain ID	SRA accession number	Assembly number	BioSample accession	WGS accession number	GC%	Illumina reads	Genome size	Contigs	*N*_50_	Serovar
3	SRR29437350	GCA_041361415.1	SAMN40140373	JBEJWJ000000000	52.12	3,625,392	4,973,597	85	148,750	*S*. Infantis
4	SRR29437349	GCA_041361515.1	SAMN40140374	JBEJWK000000000	52.14	3,389,570	4,952,536	91	115,763	*S*. Infantis
5	SRR29437338	GCA_041361535.1	SAMN40140375	JBEJWL000000000	52.14	2,493,086	4,952,947	83	148,750	*S*. Infantis
10	SRR29437327	GCA_041361555.1	SAMN40140376	JBEJWM000000000	52.14	3259866	4,955,234	74	182,593	*S*. Infantis
11	SRR29437316	GCA_041361575.1	SAMN40140377	JBEJWN000000000	52.14	1,668,738	4,952,159	79	152,177	*S*. Infantis
12	SRR29437305	GCA_041361595.1	SAMN40140378	JBEJWO000000000	52.14	1,932,474	4,954,298	78	170,752	*S*. Infantis
13	SRR29437294	GCA_041361635.1	SAMN40140379	JBEJWP000000000	52.12	3,621,548	4,971,086	90	135,876	*S*. Infantis
15	SRR29437283	GCA_041361615.1	SAMN40140380	JBEJWQ000000000	52.13	3,212,802	4,971,117	112	96,668	*S*. Infantis
16	SRR29437272	GCA_041361655.1	SAMN40140381	JBEJWR000000000	52.14	3,053,702	4,975,298	92	131,358	*S*. Infantis
17	SRR29437261	GCA_041361675.1	SAMN40140382	JBEJWS000000000	52.12	3,521,974	4,973,499	88	143,596	*S*. Infantis
18	SRR29437348	GCA_041361705.1	SAMN40140383	JBEJWT000000000	52.12	3,535,992	4,974,434	79	148,750	*S*. Infantis
19	SRR29437347	GCA_041361695.1	SAMN40140384	JBEJWU000000000	52.13	3,278,932	4,976,008	85	135,876	*S*. Infantis
20	SRR29437346	GCA_041361735.1	SAMN40140385	JBEJWV000000000	52.13	3,325,274	4,975,105	93	99,941	*S*. Infantis
21	SRR29437345	GCA_041361755.1	SAMN40140386	JBEJWW000000000	52.14	1,148,962	4,944,342	70	161,263	*S*. Infantis
22	SRR29437344	GCA_041361775.1	SAMN40140387	JBEJWX000000000	52.13	4,103,758	4,934,163	106	94,639	*S*. Infantis
23	SRR29437343	GCA_041361815.1	SAMN40140388	JBEJWY000000000	52.12	1,751,682	4,943,505	78	152,216	*S*. Infantis
27	SRR29437342	GCA_041361795.1	SAMN40140389	JBEJWZ000000000	52.13	2,769,452	4,969,523	81	145,615	*S*. Infantis
28	SRR29437341	GCA_041361835.1	SAMN40140390	JBEJXA000000000	52.12	1,688,676	4,937,013	77	145,958	*S*. Infantis
29	SRR29437340	GCA_041361855.1	SAMN40140391	JBEJXB000000000	52.13	4,277,524	4,945,099	81	145,958	*S*. Infantis
30	SRR29437339	GCA_041361875.1	SAMN40140392	JBEJXC000000000	52.15	3,337,174	4,933,065	139	84,552	*S*. Infantis
33	SRR29437337	GCA_041361915.1	SAMN40140393	JBEJXD000000000	52.18	2,764,298	4,953,628	147	68,921	*S*. Infantis
34	SRR29437336	GCA_041361895.1	SAMN40140394	JBEJXE000000000	52.16	3,216,484	4,960,326	148	69,131	*S*. Infantis
35	SRR29437335	GCA_041361935.1	SAMN40140395	JBEJXF000000000	52.14	2,418,514	4,968,299	108	97,715	*S*. Infantis
36	SRR29437334	GCA_041361995.1	SAMN40140396	JBEJXG000000000	52.14	2,040,996	4,933,008	117	91,945	*S*. Infantis
37	SRR29437333	GCA_041362035.1	SAMN40140397	JBEJXH000000000	52.00	143,880	4,696,356	1800	3,781	*S*. Infantis
40	SRR29437332	GCA_041361955.1	SAMN40140398	JBEJXI000000000	52.08	258,002	4,921,257	1388	5,471	*S*. Infantis
43	SRR29437331	GCA_041361975.1	SAMN40140399	JBEJXJ000000000	52.06	193,946	4822661	1677	4,185	*S*. Infantis
45	SRR29437330	GCA_041362015.1	SAMN40140400	JBEJXK000000000	52.12	363,746	4,969,487	143	71,382	*S*. Infantis
46	SRR29437329	GCA_041362055.1	SAMN40140401	JBEJXL000000000	52.18	111,742	4,288,931	2589	2,102	*S*. Infantis
51	SRR29437328	GCA_041362095.1	SAMN40140402	JBELNA000000000	51.90	111280	3,700,466	3100	1,341	*S*. Infantis
54	SRR29437326	GCA_041362115.1	SAMN40140403	JBEJXM000000000	52.07	169,624	4,662,264	2570	2,330	*S*. Infantis
57	SRR29437325	GCA_041362075.1	SAMN40140404	JBEJXN000000000	52.11	137,584	4,463,721	1748	3,634	*S*. Infantis
60	SRR29437324	GCA_041362135.1	SAMN40140405	JBEJXO000000000	52.07	208,596	4,807,947	2108	3,168	*S*. Infantis
61	SRR29437323	GCA_041362195.1	SAMN40140406	JBEJXP000000000	52.03	195,480	4,947,585	1128	7,020	*S.* Typhimurium
62	SRR29437322	GCA_041362175.1	SAMN40140407	JBEJXQ000000000	52.12	308,782	4,934,452	202	56,186	*S*. Infantis
63	SRR29437321	GCA_041362215.1	SAMN40140408	JBEJXR000000000	52.06	279,858	5,044,564	370	28,064	*S.* Typhimurium
64	SRR29437320	GCA_041362155.1	SAMN40140409	JBEJXS000000000	52.09	378,672	5,035,141	159	60,780	*S*. Infantis
65	SRR29437319	GCA_041362235.1	SAMN40140410	JBEJXT000000000	52.13	332,358	4,934,456	231	35,849	*S*. Infantis
66	SRR29437318	GCA_041362285.1	SAMN40140411	JBEJXU000000000	52.23	295,970	5,257,206	1013	9,317	*S*. Infantis
67	SRR29437317	GCA_041362315.1	SAMN40140412	JBEJXV000000000	52.27	195,286	4,153,575	3765	1,213	*S*. Infantis
68	SRR29437315	GCA_041362255.1	SAMN40140413	JBEJXW000000000	52.14	532,702	4,976,347	125	87,736	*S*. Infantis
69	SRR29437314	GCA_041362275.1	SAMN40140414	JBEJXX000000000	52.14	581,614	4,967,065	147	71,313	*S*. Infantis
72	SRR29437313	GCA_041362335.1	SAMN40140415	JBEJXY000000000	52.24	149,174	4,715,903	1560	4,315	*S*. Infantis
73	SRR29437312	GCA_041362355.1	SAMN40140416	JBEJXZ000000000	52.13	178,594	4,849,803	870	9,153	*S*. Infantis
79	SRR29437311	GCA_041362415.1	SAMN40140417	JBEJYA000000000	52.07	156,936	4,515,488	2915	1,936	*S*. Infantis
80	SRR29437310	GCA_041362435.1	SAMN40140418	JBEJYB000000000	52.26	114,472	4,001,928	1866	3,083	*S*. Enteritidis
103	SRR29437309	GCA_041362395.1	SAMN40140419	JBEJYC000000000	52.12	754,286	4,943,029	79	124,954	*S*. Infantis
107	SRR29437308	GCA_041362375.1	SAMN40140420	JBEJYD000000000	52.14	1,341,592	4,942,047	122	83,343	*S*. Infantis
114	SRR29437307	GCA_041362495.1	SAMN40140421	JBEJYE000000000	52.14	934,932	4,935,039	146	75,813	*S*. Infantis
118	SRR29437306	GCA_041362455.1	SAMN40140422	JBEJYF000000000	52.15	1,304,236	4,960,060	127	81,983	*S*. Infantis
119	SRR29437304	GCA_041362475.1	SAMN40140423	JBEJYG000000000	52.15	1,360,738	4,940,024	148	58,819	*S*. Infantis
120	SRR29437303	GCA_041362515.1	SAMN40140424	JBEJYH000000000	52.14	803,724	4,938,969	151	70,114	*S*. Infantis
144	SRR29437302	GCA_041362555.1	SAMN40140425	JBEJYI000000000	52.11	1,008,628	4,709,537	160	52,615	*S*. Enteritidis
146	SRR29437301	GCA_041362535.1	SAMN40140426	JBEJYJ000000000	52.16	1,315,362	4,945,474	146	62,836	*S*. Infantis
151	SRR29437300	GCA_041362575.1	SAMN40140427	JBEJYK000000000	52.13	1,389,954	4,952,274	77	148,750	*S*. Infantis
181	SRR29437299	GCA_041362595.1	SAMN40140428	JBEJYL000000000	52.03	1,450,532	4,986,725	106	92,021	*S*. Infantis
184	SRR29437298	GCA_041362615.1	SAMN40140429	JBEJYM000000000	52.12	913,334	4,969,777	90	131,232	*S*. Infantis
195	SRR29437297	GCA_041362675.1	SAMN40140430	JBEJYN000000000	52.16	1,312,506	4,678,638	137	67,740	*S*. Enteritidis
199	SRR29437296	GCA_041362635.1	SAMN40140431	JBEJYO000000000	52.03	870,368	4,984,231	95	115,763	*S*. Infantis
219	SRR29437295	GCA_041362695.1	SAMN40140432	JBEJYP000000000	52.11	790,954	4,925,748	75	150,722	*S*. Typhimurium
228	SRR29437293	GCA_041362655.1	SAMN40140433	JBEJYQ000000000	52.16	350,432	4,670,028	207	48,391	*S*. Enteritidis
233	SRR29437292	GCA_041362715.1	SAMN40140434	JBEJYR000000000	52.13	581,892	4,685,420	53	172,821	*S*. Enteritidis
320	SRR29437291	GCA_041362775.1	SAMN40140435	JBEJYS000000000	52.05	1,070,012	4,664,195	37	247,671	*S*. Enteritidis
342	SRR29437290	GCA_041362735.1	SAMN40140436	JBEJYT000000000	52.13	654,628	4,946,429	86	148,750	*S*. Infantis
343	SRR29437289	GCA_041362755.1	SAMN40140437	JBEJYU000000000	52.14	1,732,278	4,970,079	124	77,300	*S*. Infantis
344	SRR29437288	GCA_041362795.1	SAMN40140438	JBEJYV000000000	52.13	840,176	4,949,736	85	127,802	*S*. Infantis
348	SRR29437287	GCA_041362815.1	SAMN40140439	JBEJYW000000000	52.05	1,404,594	4,719,825	56	165,658	*S*. Enteritidis
359	SRR29437286	GCA_041362855.1	SAMN40140440	JBEJYX000000000	52.10	1,179,114	4,920,699	89	149,554	*S*. Infantis
361	SRR29437285	GCA_041362835.1	SAMN40140441	JBEJYY000000000	52.12	757,686	4,967,376	92	121,409	*S*. Infantis
362	SRR29437284	GCA_041362875.1	SAMN40140442	JBEJYZ000000000	52.13	1,660,700	4,964,029	97	120,857	*S*. Infantis
365	SRR29437282	GCA_041362895.1	SAMN40140443	JBEJZA000000000	52.12	3,637,956	4,970,592	72	183,643	*S*. Infantis
371	SRR29437281	GCA_041362915.1	SAMN40140444	JBEJZB000000000	52.14	3,342,994	4,966,746	80	145,958	*S*. Infantis
373	SRR29437280	GCA_041362935.1	SAMN40140445	JBEJZC000000000	52.14	2,869,498	4,961,442	82	181,393	*S*. Infantis
387	SRR29437279	GCA_041362955.1	SAMN40140446	JBEJZD000000000	52.05	2,907,052	4,732,606	57	229,432	*S*. Enteritidis
388	SRR29437278	GCA_041362975.1	SAMN40140447	JBEJZE000000000	52.11	2,232,620	4,990,363	113	152,176	*S*. Infantis
416	SRR29437277	GCA_041363015.1	SAMN40140448	JBEJZF000000000	52.13	2,172,432	4,936,585	66	171,818	*S*. Typhimurium
422	SRR29437276	GCA_041362995.1	SAMN40140449	JBEJZG000000000	52.13	1,068,986	4,699,842	55	220,671	*S*. Enteritidis
423	SRR29437275	GCA_041363035.1	SAMN40140450	JBEJZH000000000	52.14	961,214	4,956,732	84	170,434	*S*. Infantis
427	SRR29437274	GCA_041363055.1	SAMN40140451	JBEJZI000000000	52.05	1,352,250	4,723,628	42	227,910	*S*. Enteritidis
429	SRR29437273	GCA_041363075.1	SAMN40140452	JBEJZJ000000000	52.13	1,866,278	4,958,561	77	181,393	*S*. Infantis
431	SRR29437271	GCA_041363095.1	SAMN40140453	JBEJZK000000000	52.13	1,076,040	4,959,763	72	170,895	*S*. Infantis
432	SRR29437270	GCA_041363135.1	SAMN40140454	JBEJZL000000000	52.13	2,657,164	4,957,223	80	127,689	*S*. Infantis
435	SRR29437269	GCA_041363115.1	SAMN40140455	JBEJZM000000000	52.06	1,287,448	4,724,134	39	243,706	*S*. Enteritidis
438	SRR29437268	GCA_041363155.1	SAMN40140456	JBEJZN000000000	52.12	1,796,414	4,978,483	103	170,757	*S*. Infantis
440	SRR29437267	GCA_041363175.1	SAMN40140457	JBEJZO000000000	52.11	1,609,916	5,029,439	168	161,379	*S*. Infantis
441	SRR29437266	GCA_041363195.1	SAMN40140458	JBEJZP000000000	52.13	2,268,396	4,896,968	70	171,226	*S*. Typhimurium
443	SRR29437265	GCA_041363255.1	SAMN40140459	JBEJZQ000000000	52.11	1,968,724	4,924,331	77	116,767	*S*. Typhimurium
456	SRR29437264	GCA_041363215.1	SAMN40140460	JBEJZR000000000	52.11	1,427,214	4,919,318	82	149,876	*S*. Typhimurium
458	SRR29437263	GCA_041363235.1	SAMN40140461	JBEJZS000000000	52.11	4,211,276	4,923,653	80	116,772	*S*. Typhimurium
460	SRR29437262	GCA_041363295.1	SAMN40140462	JBEJZT000000000	52.10	3,628,772	4,921,529	84	150,252	*S*. Typhimurium
463	SRR29437260	GCA_041363275.1	SAMN40140463	JBEJZU000000000	52.12	3,268,104	4,973,621	83	152,177	*S*. Infantis
464	SRR29437259	GCA_041363335.1	SAMN40140464	JBEJZV000000000	52.05	3,023,108	4,721,719	41	220,670	*S*. Enteritidis
474	SRR29437258	GCA_041363395.1	SAMN40140465	JBEJZW000000000	52.05	2,814,460	4,720,180	43	219,176	*S*. Enteritidis
477	SRR29437257	GCA_041363355.1	SAMN40140466	JBEJZX000000000	52.04	2,032,360	4,985,231	75	143,999	*S*. Typhimurium

## Data Availability

The data sets generated for this study are available in the NCBI repository under BioProject PRJNA1080051. The Whole Genome Shotgun project has been deposited in DDBJ/ENA/GenBank under the accession numbers JBEJWJ000000000 to JBELNA000000000. The version described in this paper is JBEJWJ010000000 to JBELNA010000000. Raw sequence reads have been submitted to the Sequence Read Archive (SRA) and can be found in Table 1.

## References

[1] Punchihewage-Don AJ, Ranaweera PN, Parveen S. 2024. Defense mechanisms of Salmonella against antibiotics: a review. Front Antibiot 3:1448796. doi:10.3389/frabi.2024.1448796 39816264 PMC11731628

[2] Punchihewage-Don AJ, Hawkins J, Adnan AM, Hashem F, Parveen S. 2022. The outbreaks and prevalence of antimicrobial resistant Salmonella in poultry in the United States: an overview. Heliyon 8:e11571. doi:10.1016/j.heliyon.2022.e11571 36406693 PMC9668525

[3] Elbashir SM, Adnan AM, Bowers J, DePaola A, Jahncke M, Punchihewage-Don AJ, Da Silva LV, Hashem F, Parveen S. 2023. Antimicrobial resistance, virulence properties and genetic diversity of Salmonella Typhimurium recovered from domestic and imported seafood. Pathogens 12:897. doi:10.3390/pathogens12070897 37513743 PMC10384935

[4] CDC. 2024. Salmonella. Available from: https://www.cdc.gov/salmonella/index.html. Retrieved 18 Sep 2024.

[5] CDC. 2024. Chicken and food poisoning. Available from: https://www.cdc.gov/food-safety/foods/chicken.html. Retrieved 7 Sep 2024.

[6] FDA. 2023. Get the facts about Salmonella, on U.S. Food and Drug Administration. Available from: https://www.fda.gov/animal-veterinary/animal-health-literacy/get-facts-about-salmonella. Retrieved 22 Aug 2024.

[7] Punchihewage-Don AJ, Parveen S, Schwarz J, Hamill L, Nindo C, Hall P, Vimini B. 2021. Efficacy and quality attributes of antimicrobial agent application via a commercial electrostatic spray cabinet to inactivate Salmonella on chicken thigh meat. J Food Prot 84:2221–2228. doi:10.4315/JFP-21-206 34410413

[8] Punchihewage-Don AJ, Schwarz J, Diria A, Bowers J, Parveen S. 2023. Prevalence and antibiotic resistance of Salmonella in organic and non-organic chickens on the Eastern Shore of Maryland, USA. Front Microbiol 14:1272892. doi:10.3389/fmicb.2023.1272892 38239721 PMC10794514

[9] USDA-FSIS. 2023. Isolation and identification of Salmonella from meat, poultry, pasteurized egg, siluriformes (fish) products and carcass and environmental sponges. Available from: https://www.fsis.usda.gov/news-events/publications/microbiology-laboratory-guidebook. Retrieved 19 Aug 2024.

[10] Punchihewage-Don AJ, Hasan NA, Rashed SM, Parveen S. 2023. Microbiome analysis of organic and conventional chickens processed using whole carcass enrichment and rinse methods. J Food Prot 86:100176. doi:10.1016/j.jfp.2023.100176 37805044

[11] Clinical and Laboratory Standards Institute (CLSI. 2016. Performance standards for antimicrobial susceptibility testing. 26th ed. Clinical and Laboratory Standards Institute, Pennsylvania, USA.

[12] Elbashir S, Jahncke M, DePaola A, Bowers J, Schwarz J, Punchihewage-Don AJ, Min B, Rippen T, Parveen S. 2023. Prevalence and abundance of bacterial pathogens of concern in shrimp, catfish and tilapia obtained at retail stores in Maryland, USA. Pathogens 12:187. doi:10.3390/pathogens12020187 36839458 PMC9963610

[13] Gangiredla J, Rand H, Benisatto D, Payne J, Strittmatter C, Sanders J, Wolfgang WJ, Libuit K, Herrick JB, Prarat M, Toro M, Farrell T, Strain E. 2021. GalaxyTrakr: a distributed analysis tool for public health whole genome sequence data accessible to non-bioinformaticians. BMC Genomics 22:114. doi:10.1186/s12864-021-07405-8 33568057 PMC7877046

[14] Zhang S, Yin Y, Jones MB, Zhang Z, Deatherage Kaiser BL, Dinsmore BA, Fitzgerald C, Fields PI, Deng X. 2015. Salmonella serotype determination utilizing high-throughput genome sequencing data. J Clin Microbiol 53:1685–1692. doi:10.1128/JCM.00323-15 25762776 PMC4400759

[15] Bharat A, Petkau A, Avery BP, Chen JC, Folster JP, Carson CA, Kearney A, Nadon C, Mabon P, Thiessen J, Alexander DC, Allen V, El Bailey S, Bekal S, German GJ, Haldane D, Hoang L, Chui L, Minion J, Zahariadis G, Domselaar GV, Reid-Smith RJ, Mulvey MR. 2022. Correlation between phenotypic and In Silico detection of antimicrobial resistance in Salmonella enterica in Canada using Staramr. Microorganisms 10:292. doi:10.3390/microorganisms10020292 35208747 PMC8875511

[16] Chen L, Zheng D, Liu B, Yang J, Jin Q. 2016. VFDB 2016: hierarchical and refined dataset for big data analysis—10 years on. Nucleic Acids Res 44:D694–D697. doi:10.1093/nar/gkv1239 26578559 PMC4702877

[17] Yoon SH, Park YK, Kim JF. 2015. PAIDB v2.0: exploration and analysis of pathogenicity and resistance islands. Nucleic Acids Res 43:D624–30. doi:10.1093/nar/gku985 25336619 PMC4384037

